# Inter-clinician diagnostic agreement of shock etiology: a multicenter observational study

**DOI:** 10.1007/s13755-025-00423-w

**Published:** 2026-01-12

**Authors:** Lauren M. Janczewski, Carolyn J. Hu, Tiannan Zhan, Ravi Garg, John Slocum, Al’ona Furmanchuk, Andrew Berry, Nandita Nadig, Jeff Huml, Laeeq Shamshuddin, Yuriy Moklyak, Laura J. Davidson, Sherry Chou, Abbas AlQamari, Emilie Powell, Bruce Ankenman, Jane L. Holl, Anne Stey

**Affiliations:** 1 Department of Surgery, Feinberg School of Medicine, Northwestern University, 676 N. St. Clair, Suite 650, Chicago, IL 60611 USA; 2 Department of Medicine, Feinberg School of Medicine, Northwestern University, Chicago, IL USA; 3 Department of Medical Social Science, Feinberg School of Medicine, Northwestern University, Chicago, IL USA; 4 Department of Neurology, Feinberg School of Medicine, Northwestern University, Chicago, IL USA; 5 Department of Anesthesiology, Feinberg School of Medicine, Northwestern University, Chicago, IL USA; 6 Department of Emergency Medicine, Feinberg School of Medicine, Northwestern University, Chicago, IL USA; 7 Department of Industrial Engineering and Management Studies, McCormick School of Engineering, Northwestern University, Chicago, IL USA; 8 Center for Healthcare Delivery Science and Innovation, University of Chicago, Chicago, IL USA

**Keywords:** Shock, Intensive care unit, Diagnostic agreement, Machine learning

## Abstract

**Purpose:**

We sought to (1) quantify lack of inter-clinician diagnostic agreement of shock etiology and (2) predict patients without complete inter-clinician diagnostic agreement of shock etiology.

**Methods:**

This multicenter retrospective, cohort study identified patients evaluated by two or more clinicians who documented a shock diagnosis from 2018 to 2023 across intensive care units (ICU) at 9 acute care hospitals. Shock etiology was abstracted using regular expression from clinician notes in the electronic health record then was made into a 9-dimensional vector representing 9 different shock etiologies. Inter-clinician diagnostic agreement of these vectors was calculated for each patient using Cosine Similarity Scores. Measure of agreement was based on cosine similarity of etiology vectors, not clinical adjudication. Patients without complete inter-clinician diagnostic agreement (Cosine Similarity Score < 1) were compared to patients with diagnostic agreement. Machine learning models were tested to predict patients without complete inter-clinician diagnostic agreement.

**Results:**

Of 7302 patients, 1327 (18.2%) never had complete inter-clinician diagnostic agreement. Patients without diagnostic agreement were more often Black (20.5 vs 16.2%, *p* = 0.011), with more comorbidities (Elixhauser Comorbidity Index > 10; 39.1 vs 31.6%, *p* < 0.001), and Sequential Organ Failure Assessment (SOFA) score > 15 (12.1 vs 7.6%, *p* < 0.001). Patients without diagnostic agreement less frequently had improvements in SOFA scores between ICU days 0 and 4 (34.7 vs 41.9%, *p* < 0.001), and more often died in-hospital (41.5 vs. 27.6%, *p* < 0.001). Machine learning models that most accurately predicted patients without diagnostic agreement were logistic regression (Accuracy: 0.8597, F1-Score: 0.9117, AUC-ROC: 0.9257), random forest (Accuracy: 0.8658, F1-Score: 0.9201, AUC-ROC: 0.9255), and gradient boosting (Accuracy: 0.8515, F1-Score: 0.9138, AUC-ROC: 0.9227).

**Conclusion:**

Patients without complete inter-clinician diagnostic agreement of shock etiology can be successfully predicted.

**Supplementary Information:**

The online version contains supplementary material available at 10.1007/s13755-025-00423-w.

## Introduction

Shock is a prevalent, lethal condition triggering the hospitalization of 1.3 million patients annually in the United States [1]. The underlying cause, or shock etiology is never identified in an estimated 70,000 cases annually [2]. Patient diagnosis commonly requires input from many clinicians of different disciplines to determine the primary shock etiology and best treatment plan [3, 4]. Mortality rates range between 30 and 50% and depend largely on the shock etiology diagnosis as well as treatment timeliness [2, 3, 5–7]. Shock is one of the costliest conditions to treat costing $31,000–$68,000 per admission [8].

Prior research has focused on identifying whether shock was present [1], especially for common specific shock etiologies, such as septic shock. There is little literature on establishing the underlying shock etiology diagnosis. Many clinicians are involved in the diagnosis and treatment of shock. Inter-clinician diagnostic agreement may help drive more timely treatment, and lower mortality [9]. The rate of inter-clinician diagnostic agreement of shock etiology, and whether patients without inter-clinician diagnostic agreement of shock etiology can be predicted have not been studied.

The study objective was to characterize inter-clinician diagnostic agreement of shock etiology. The first aim sought to quantify the frequency of patients without inter-clinician diagnostic agreement of shock etiology. The second aim was to build a machine learning model to predict which patients would not have inter-clinician diagnostic agreement of shock etiology. We hypothesized that patients without inter-clinician diagnostic agreement would experience higher in-hospital mortality, and that machine learning models could successfully predict which patients would not have complete inter-clinician diagnostic agreement of shock etiology.

## Methods

### Study design and setting

This multicenter observational, retrospective, cohort study identified patients with shock between January 1st, 2018, and December 31st, 2023, within an integrated academic urban healthcare system located in the Midwestern United States comprised of 9 acute care hospitals. These hospitals included one academic medical center, seven community hospitals, and one critical access hospital. This study was approved by the Northwestern University Institutional Review Board under study number STU00218401. The transparent reporting of a multivariable prediction model for individual prognosis or diagnosis (TRIPOD) reporting guidelines was followed. All procedures were followed in accordance with the Helsinki Declaration of 1975.

### Data source

Electronic Health Record (EHR) structured and unstructured data were obtained from the healthcare system Enterprise Data Warehouse (EDW). The systemwide integrated EDW captured all inpatient encounters across all hospitals in the healthcare system from 2018 until 2023. This data source has been used to successfully identify the presence of shock as well as the shock phenotype among injured patients in a variety of geographic context (urban, suburban and rural) and hospital settings (academic medical center, community hospitals and critical access hospitals) [10].

### Study population

Eligibility criteria were adult patients greater than or equal to 18 years of age, hospitalized in an intensive care unit (ICU) at any one of the 9 acute care hospitals between 2018 and 2023 with shock. Shock was defined using both structured (vitals and labs) and unstructured (notes) data. Shock patient encounters were first identified with structured data, specifically a systolic blood pressure measurement of less than 90 mmHg, AND with at least one new organ failure as measured by a new elevation in the Sequential Organ Failure Assessment (SOFA) score from the last recorded measurement [11, 12]. Among these patient encounters, the shock cohort was further narrowed to include only patient encounters with “shock” documented in history and physical, progress, or consult notes using regular expression from the same day (day 0) as systolic blood pressure measurement of less than 90 mmHg and new elevation in the SOFA score. Patient encounters with notes only using negation terms were excluded. Patient encounters with less than two clinician notes documenting “shock” were excluded (eFig. 1) as two or more notes on any one day were needed to determine inter-clinician diagnostic agreement.

### Shock etiology diagnosis

Shock etiology diagnosis was a priori collapsed into nine different shock etiologies Septic, Cardiogenic, Hypovolemic, Adrenal, Neurogenic, Undifferentiated, Obstructive, Anaphylactic, Post-procedural) based on clinical experience and prior literature.[13] Regular expressions were applied  to all unstructured data documented in clinician history and physical, progress, or consult notes from the first day of the systolic blood pressure measurement of less than 90 mmHg and new elevation in the SOFA score (called day 0) through day four. A daily clinician-specific shock etiology diagnosis variable was created for each clinician note [14]. Septic shock was identified by presence of the string value “Sepsis”, “Septic”, and “Septic Shock” without negation statements. Cardiogenic shock was identified by presence of the string value “Cardiac Shock”, “Cardiogenic”, “Cardiogenic Shock” without negation statement. Hypovolemic shock was identified by presence of the string value “hypovolemic”, “hypovolemia”, “hypovolemic shock”, “hemorrhage”, “hemorrhagic shock” and “traumatic shock” without negation statement. Adrenal shock was identified by presence of the string value “adrenal shock”, and “adrenal insufficiency” without negation statement. Neurogenic shock was identified by presence of the string value “spinal shock”, and “neurogenic shock” without negation statement. Undifferentiated shock was identified by the presence of the string value “undifferentiated shock”, “shock NOS”, and “multifactorial shock” without negation statement. Obstructive shock was identified by presence of the string value “obstructive shock”, “cardiac tamponade”, “tamponade”, “tension pneumothorax” and “tension pneumo” without negation statement. Anaphylactic shock was identified by the presence of the string value “anaphylaxis”, “anaphylactic” and “anaphylactic shock” without negation statement. Post-procedural shock was identified by presence of the string value “post-procedure shock”, and “post-procedural shock” without negation statement. Shock etiology diagnosis was abstracted from each clinician note in the electronic health record, for each patient, on each day 0 through day 4. We manually reviewed all occurrences to identify if ambiguous or overlapping terms were present. “Undifferentiated Shock” was the ambiguous term most frequently used, and we created a separate vector (of the nine) labeled as such. Overlapping terms, such as the different shock diagnoses, in the same note or by the same clinician in a later note each day were treated as discrete observations generating an additional vector for each separate shock diagnosis. Additionally, we manually reviewed all observations and ensured that context-sensitive language was not playing any role here. A total of 395 patients were reviewed with ambiguous phrases adjudicated by two clinicians (LJ, AMS). Ambiguous or mixed phrases (e.g., “distributive shock,” “shock of unclear etiology”) were mapped onto the unknown category. When two different forms of shock were hypothesized then (e.g., “mixed septic and cardiogenic shock,”), the clinician note was annotated for both Septic and Cardiogenic shock.

### Outcome of interest

The primary outcome of interest was inter-clinician diagnostic agreement of shock etiology diagnosis. Each clinician note, each dayfor each patient was interpreted as a nine-dimensional vector of binary values (i.e., 0 = not mentioned, 1 = mentioned) indicating mention of each of the nine shock etiologies above. Each clinicians’ daily diagnosis documented in their notes were compared to every other clinician’s daily diagnosis from days 0 to 4. The agreement between the clinicians’ shock etiology diagnoses was quantified using cosine similarity scores for nine-dimensional vectors from days 0 to 4. Cosine similarly measures the alignment of two vectors, with scores ranging from 0 to 1; a score of 1 indicates complete alignment, representing complete inter-clinician diagnostic agreement [15]. Patients were categorized based on whether they ever achieved a daily average cosine similarity score of 1 during the first four days of shock. Two near-perfect cosign similarity scores thresholds (≥ 0.95; ≥ 0.90) were also tested in sensitivity analyses. The measure of agreement was based on cosine similarity of etiology vectors, not on direct clinical adjudication. This study sought to understand the patient and clinician characteristics associated with not having complete inter-clinician diagnostic agreement of shock etiology, and to evaluate the ability of machine learning methods to predict which patients would never achieve inter-clinician agreement.

### Independent variables

Independent variables were patient demographics, clinical factors, clinical course, and clinician characteristics. Patient demographics included age in years which was categorized into five groups, 50 and under, 51–60, 61–70, 71–80 and 81 and older. Sex was categorized as male and female. Race was categorized into seven groups as White, Black/African American, Asian, Native Hawaiian/Pacific Islander, American Indian/Alaska Native, Other and Unknown. Ethnicity was categorized into three groups as Hispanic, not Hispanic, and Unknown. Insurance was categorized into four groups as Medicare, Medicaid, Private and Self-pay. Area deprivation index (ADI) rank was calculated based on the patients address [16]. A larger number suggested patient lived in a neighborhood with greater socioeconomic deprivation. The ADI rank at the state was categorized into four groups as 1–2, 3–5, 6–10 and unknown, as well as categorized by the national rank into five groups as < 25, 25–49, 50–74, 75–100 and unknown.

Clinical features were SOFA score, Sepsis Score, Shock Index, shock etiology, Elixhauser comorbidity index, and Body Mass Index. Contextual clinical features were whether the first shock diagnosis occurred during the weekday or weekend, whether the ICU admission was on the weekday or weekend, and number of unique clinicians who wrote a note on the patient in the first 4 days of shock. The first recorded SOFA score was calculated from laboratory and clinical data for each patient upon shock presentation and was categorized into five groups as 0–6, 7–12, 13–15, 16–24, no SOFA score [17]. The sepsis score was categorized into six groups as 0, 1, 2, 3, >  = 4, not applicable [18]. The shock index was calculated by dividing heart rate by systolic blood pressure then categorized in five groups as < 0.5, 0.5–0.7, 0.7–1.0, > 1.0, unknown [19]. The nine shock etiologies were described above. Elixhauser comorbidity index was calculated from the International Classification of Disease Codes and categorized into four groups: 0–5, 5–6, 7–10 and > 10. Body mass index (BMI) was calculated as weight (kg) divided by height (m^2^) and categorized into six groups: < 18.5, 18.5–24.9, 25.0–29.9, 30.0–34.9, ≥ 35, and unknown. ICU admission day and first diagnosis of shock day were each categorized as weekday or weekend. The daily note count, and unique clinician count per patient were calculated and treated as continuous variables.

Clinical course characteristics were SOFA score changes, treatments administered, ICU mortality and discharge disposition. SOFA score change was calculated as the difference between day 0 and day 4 then was categorized into four groups: improved, no change/worse, no SOFA score, and only one value recorded. Treatments included time to first intravenous bolus fluids (normal saline, lactated ringers or plasmalyte) and was categorized as < 24, 24–48, 49–72, 73–96, and > 96 h. The administration of intravenous fluids was recorded dichotomously by the day of shock presentation (days 0–4). Antifungal administration (micafungin, fluconazole, amphotericin) was categorzied as a dichotomous variable. Time to first antibiotics (eTable 1) was categorized similarly to intravenous fluids. Anticoagulation infusions (heparin, bivalirudin, argatroban) and diuretics (furosemide, bumetanide) were categorized dichotomously. Time to first lab draw, echocardiogram, and surgical procedure were categorized into six group variable: < 24, 24–48, 49–72, 73–96, and > 96 h. Procedures such as arterial line placement, cardiac catheterization, central venous catheter placement, and continuous renal replacement therapy were recorded as dichotomous variable . ICU mortality was a dichotomous outcome Discharge disposition was categorized as transfer to another acute care hospital, acute inpatient rehabilitation, long-term care facility, home, death, or unknown. Clinician characteristics included clinician type (e.g., physician, advanced practice provider) and specialty (e.g., cardiology).

### Statistical analysis

The rate of patients which never had complete inter-clinician diagnostic agreement on any of the four days was calculated. Differences in patient demographics, clinical features, and clinical course between patients with complete inter-clinician diagnostic agreement and without were compared using chi-squared tests of independence. Clinician characteristics were compared between notes with and without complete diagnostic agreement using chi-squared tests, with statistical significance set at *p*-value < 0.05. Unadjusted analyses were performed in R version 4.3.0.

Machine learning models were developed to predict patients without complete inter-clinician diagnostic agreement on shock etiology. Data were split into 80% training and 20% validation cohorts. On the 80% training data, we performed fivefold cross-validation to tune the parameters of each of the six machine learning models. The six machine learning models tuned were logistic regression, Random Forest, Decision Trees, Gradient Boosting, K-Nearest Neighbor (KNN), and Support Vector Machine (SVM) algorithms. The best-performing parameters from cross-validation were used to train on all 80% of data to obtain the final models. These models aimed to predict patients without complete inter-clinician diagnostic agreement. Model performance was assessed using accuracy, F1 score, and the receiver operating characteristic (AUC-ROC) on the validation cohort, with metrics ranging from 0.0 to 1.0, where values closer to 1.0 indicate higher performance [20]. Calibration plots were created to compare predicted and observed probabilities for each of the six models. A perfectly calibrated model’s curve should align closely with the diagonal, indicating accurate predictions. The hyperparameters that were tuned were the AUC-ROC and F1 score using the fivefold cross-validation on the 80% training set only. The reported performance metrics (accuracy, F1, AUC-ROC) and calibration curves were all based on the 20% held-out validation cohort. Two sensitivity analyses were conducted to evaluate overly stringent threshold for inter-clinician agreement and model evaluation was performed for near-perfect cosign similarity scores thresholds (≥ 0.95; ≥ 0.90).

Feature importance was calculated using Random Forest algorithm to identify patient, clinical, and clinician features predictive of inter-clinician diagnostic agreement on shock etiology. Random Forest algorithm ranks feature importance in decision tree regression [21]. Analyses were performed in Python version 3.10.9.

## Results

There were 7302 unique patients admitted to the ICU with shock with documented shock by at least two different clinicians on one day  in a total of 35,411 notes. In total, 5975 (81.8%) patients had complete inter-clinician diagnostic agreement of the shock etiology at any time during the first four days of shock presentation. Conversely, 1327 (18.2%) never had complete inter-clinician diagnostic agreement of shock etiology.

Patients without complete inter-clinician diagnostic agreement were more often Black (20.5 vs 16.2%, *p* = 0.011), compared to patients with complete inter-clinician diagnostic agreement (Table 1). Patients without complete inter-clinician diagnostic agreement more often had a SOFA score greater than 15 (12.1 vs 7.6%, *p* < 0.001) (Table 2). Patients without complete inter-clinician diagnostic agreement more often had an Elixhauser Comorbidity Index greater than 10 (39.1 vs 31.6%, *p* < 0.001).

**Table 1:** Shock patient demographics by whether there was complete inter-clinician diagnostic agreement of shock etiology captured in electronic health record clinician notes in 9 acute care hospitals from 2018 to 2023

	Never had total agreement, *N* (%)	Evidence of total agreement, *N* (%)	*p*-value
	*N* = 1327 (18.2)	*N* = 5975 (81.8)	
Age (years)			
50 and under	171 (12.9)	833 (14.0)	0.634
51 to 60	169 (12.7)	820 (13.8)	
61 to 70	337 (25.4)	1454 (24.4)	
71 to 80	357 (26.9)	1576 (26.5)	
81 and older	293 (22.1)	1273 (21.4)	
Sex			
Male	756 (57.0)	3348 (56.0)	0.554
Female	571 (43.0)	2627 (44.0)	
Race			
White	872 (65.7)	4181 (70.0)	0.011
Black or African American	272 (20.5)	970 (16.2)	
Asian	43 (3.2)	222 (3.7)	
Native Hawaiian or Other Pacific Islander	3 (0.2)	13 (0.2)	
American Indian or Alaska Native	5 (0.4)	14 (0.2)	
Other	90 (6.8)	410 (6.9)	
Unknown, declined, unable to answer	42 (3.2)	165 (2.8)	
Ethnicity			
Hispanic, Latino, or Spanish origin	113 (8.5)	544 (9.1)	0.159
Not Hispanic, Latino, or Spanish origin	1158 (87.3)	5239 (87.7)	
Unknown, declined, unable to answer	56 (4.2)	192 (3.2)	
Insurance			
Medicare	843 (63.5)	3664 (61.3)	0.152
Medicaid	166 (12.5)	719 (12.0)	
Private	302 (22.8)	1484 (24.8)	
Self-Pay	16 (1.2)	108 (1.8)	
ADI national rank			
< 25	209 (27.4)	875 (25.0)	0.235
25–49	260 (34.0)	1307 (37.3)	
50–74	206 (27.0)	943 (26.9)	
75–100	80 (10.5)	357 (10.2)	
Unknown	9 (1.2)	23 (0.7)	
ADI state rank			
1–2	275 (36.0)	1219 (34.8)	0.04
3–5	269 (35.2)	1399 (39.9)	
6–10	211 (27.6)	864 (24.7)	
Unknown	9 (1.2)	23 (0.7)	

*BMI* body mass index, *ICU* intensive care unit, *ADI* area deprivation index

**Table 2:** Shock patient clinical features by whether there was complete inter-clinician diagnostic agreement of shock etiology captured in electronic health record clinician notes in 9 acute care hospitals from 2018 to 2023

	Never had total agreement, *N* (%)	Evidence of total agreement, *N* (%)	*p*-value
	1327 (18.2)	5975 (81.8)	
First SOFA score			
0–6 (< 2%)	509 (38.4)	2439 (40.8)	< 0.001
7–12 (0–30%)	451 (34.0)	2219 (37.1)	
13–15 (40–90%)	152 (11.5)	624 (10.4)	
16–24 (> 90%)	161 (12.1)	452 (7.6)	
No SOFA score	54 (4.1)	241 (4.0)	
Sepsis Score			
0	72 (5.4)	370 (6.2)	0.011
1	55 (4.1)	279 (4.7)	
2	69 (5.2)	358 (6.0)	
3	98 (7.4)	605 (10.1)	
> = 4	752 (56.7)	3191 (53.4)	
Unknown	281 (21.2)	1172 (19.6)	
Shock index			
< 0.5	53 (4.0)	222 (3.7)	< 0.001
0.5–0.7 (normal)	306 (23.1)	1646 (27.5)	
0.7–1.0	609 (45.9)	2820 (47.2)	
> 1.0	359 (27.1)	1286 (21.5)	
Unknown	0 (0.0)	1 (0.0)	
Shock Etiology*			
Septic Shock	1107 (83.4)	3860 (64.6)	< 0.001
Cardiogenic Shock	698 (52.6)	1389 (23.2)	< 0.001
Hypovolemic Shock	433 (32.6)	755 (12.6)	< 0.001
Adrenal Insufficiency Shock	366 (27.6)	448 (7.5)	< 0.001
Neurogenic Shock	13 (1.0)	22 (0.4)	0.007
Undifferentiated	202 (15.2)	139 (2.3)	< 0.001
Obstructive Shock	305 (23.0)	539 (9.0)	< 0.001
Anaphylactic Shock	9 (0.7)	36 (0.6)	0.901
Post-procedural Shock	2 (0.2)	0 (0.0)	0.033
Elixhauser comorbidity index			
0–5	92 (6.9)	830 (13.9)	< 0.001
5–6	201 (15.1)	1121 (18.8)	
7–10	515 (38.8)	2136 (35.7)	
> 10	519 (39.1)	1888 (31.6)	
BMI			
< 18.5	85 (6.4)	314 (5.3)	0.399
18.5–24.9	360 (27.1)	1539 (25.8)	
25.0–29.9	291 (21.9)	1370 (22.9)	
30.0–34.9	185 (13.9)	827 (13.8)	
35 and above	171 (12.9)	842 (14.1)	
Unknown	235 (17.7)	1083 (18.1)	
ICU Admission day			
Weekday	1019 (76.8)	4491 (75.2)	0.226
Weekend	308 (23.2)	1484 (24.8)	
First Diagnosis of Shock day			
Weekday	978 (73.7)	4429 (74.1)	0.775
Weekend	349 (26.3)	1546 (25.9)	
Number of unique clinicians per patient (mean (SD))	4.82 (2.79)	4.86 (2.62)	0.670

*Each row represents a separate statistical comparison. Only the number (and percent) of patients who had a diagnosis of each type of shock are presented in the rows

*SOFA* sequential organ failure assessment, *NOS* not otherwise specified, *SD* standard deviation

Patients without complete inter-clinician diagnostic agreement less frequently had improvements in SOFA scores between days 0 and 4 (34.7 vs 41.9%, *p* < 0.001) compared to patients with complete inter-clinician diagnostic agreement. Patients without complete inter-clinician diagnostic agreement more frequently had procedures such as arterial line placement (40.4 vs. 34.5%, *p* < 0.0001), central venous catheter placement (49.0 vs.41.9%, *p* < 0.0001), and continuous renal replacement therapy (21.5 vs. 12.5%, *p* < 0.0001) (Table 3). However, patients without complete inter-clinician diagnostic agreement more frequently received first antibiotic at > 96 h, (9.3 vs 11.6%, *p* = 0.002). Patients without complete inter-clinician diagnostic agreement more often died in the ICU (39.7 vs. 26.8%, *p* < 0.001) and more often died in-hospital (41.5 vs. 27.6%, *p* < 0.001). Clinician characteristics demonstrated that resident/fellow clinical notes more frequently never had complete inter-clinician diagnostic agreement (37.8 vs. 29.3%, *p* < 0.001) (Table 4).

**Table 3:** Shock patients clinical course by whether there was complete inter-clinician diagnostic agreement of shock etiology captured in electronic health record clinician notes in 9 acute care hospitals from 2018 to 2023

	Never had total agreement, *N* (%)	Evidence of total agreement, *N* (%)	*p*-value
	1327 (18.2)	5975 (81.8)	
SOFA score last-first change			
Improvement	460 (34.7)	2503 (41.9)	< 0.001
No change/Worse score	503 (37.9)	1848 (30.9)	
No score reported	54 (4.1)	241 (4.0)	
Only one value	310 (23.4)	1383 (23.1)	
Time To First IV Fluid Bolus			
< 24 h	1251 (94.3)	5668 (94.9)	0.566
25–48 h	44 (3.3)	161 (2.7)	
49–72 h	18 (1.4)	75 (1.3)	
73–96 h	5 (0.4)	37 (0.6)	
> 96 h	9 (0.7)	34 (0.6)	
Fluids Administered Day 0	1252 (94.3)	5442 (91.1)	< 0.001
Fluids Administered Day 1	1018 (76.7)	4412 (73.8)	0.033
Fluids Administered Day 2	830 (62.5)	3583 (60.0)	0.088
Fluids Administered Day 3	708 (53.4)	3134 (52.5)	0.572
Fluids Administered Day 4	634 (47.8)	2728 (45.7)	0.170
Antifungals Administered	53 (4.8)	266 (5.4)	0.453
Time To First Antibiotic			
< 24 h	763 (57.5)	3597 (60.2)	0.002
25–48 h	40 (3.0)	173 (2.9)	
49–72 h	41 (3.1)	100 (1.7)	
73–96 h	19 (1.4)	97 (1.6)	
> 96 h	154 (11.6)	553 (9.3)	
N/A	310 (23.4)	1455 (24.4)	
Anticoagulation Administered	817 (61.6)	3410 (57.1)	0.003
Diuretic Administered*			
Furosemide	401 (30.2)	1784 (29.9)	0.821
Bumetanide	358 (27.0)	1182 (19.8)	< 0.001
Time To First Lab Draw			
< 24 h	699 (52.7)	3198 (53.5)	< 0.001
25–48 h	105 (7.9)	417 (7.0)	
49–72 h	65 (4.9)	288 (4.8)	
73–96 h	57 (4.3)	214 (3.6)	
> 96 h	342 (25.8)	1256 (21.0)	
NA	59 (4.4)	602 (10.1)	
Time To First Echo			
< 24 h	21 (1.6)	64 (1.1)	0.176
25–48 h	27 (2.0)	88 (1.5)	
49–72 h	12 (0.9)	36 (0.6)	
73–96 h	6 (0.5)	24 (0.4)	
> 96 h	20 (1.5)	69 (1.2)	
N/A	1241 (93.5)	5694 (95.3)	
Time To First Surgery			
< 24 h	100 (7.5)	580 (9.7)	0.107
25–48 h	52 (3.9)	264 (4.4)	
49–72 h	39 (2.9)	199 (3.3)	
73–96 h	36 (2.7)	129 (2.2)	
> 96 h	91 (6.9)	384 (6.4)	
N/A	1009 (76.0)	4419 (74.0)	
Arterial line placement	536 (40.4)	2062 (34.5)	< 0.001
Cardiac catheterization	108 (8.1)	405 (6.8)	0.090
Central venous catheter placement	650 (49.0)	2504 (41.9)	< 0.001
Continuous renal replacement therapy	285 (21.5)	746 (12.5)	< 0.001
Interventional radiology procedure	1 (0.1)	5 (0.1)	1.000**
ICU Mortality	527 (39.7)	1601 (26.8)	< 0.001
Discharge Disposition			
Acute Care Hospital	17 (1.3)	123 (2.1)	< 0.001
Acute Inpatient Rehabilitation	117 (8.8)	581 (9.7)	
Long-term Facility	273 (20.6)	1397 (23.4)	
Home	368 (27.7)	2198 (36.8)	
Death	551 (41.5)	1648 (27.6)	
Unknown	1 (0.1)	28 (0.5)	

*Each row represents a separate statistical comparison. Only the number (and percent) of patients who received the respective procedure or medication are presented in the rows

**Fisher’s Exact test

*Hrs* hours, *IV* intravenous, *ICU* intensive care unit

**Table 4:** Shock patient clinicians’ characteristics by whether there was complete inter-clinician diagnostic agreement of shock etiology captured in electronic health record clinician notes in 9 acute care hospitals from 2018 to 2023

	Never had total agreement, *N* (%)	Evidence of total agreement, *N* (%)	*p*-value
	*N* = 6398	*N* = 29,013	
Clinician type			
Physician	2691 (42.1)	13,665 (47.1)	< 0.001
Resident/Resident Fellow	2417 (37.8)	8498 (29.3)	
Advanced Practice Provider (PA/NP)	794 (12.4)	4200 (14.5)	
Nurse	91 (1.4)	521 (1.8)	
Medical Student	68 (1.1)	334 (1.2)	
Non-shock Clinician	25 (0.4)	120 (0.4)	
Other	258 (4.0)	1414 (4.9)	
Other student/trainee	9 (0.1)	52 (0.2)	
Unknown	45 (0.7)	209 (0.7)	
Clinician Specialty			
Critical Care Medicine	364 (5.7)	2248 (7.7)	< 0.001
Internal Medicine	2764 (43.2)	12,681 (43.7)	
Cardiology	627 (9.8)	2593 (8.9)	
Surgery	575 (9.0)	2660 (9.2)	
Neurology/Neurological Surgery	428 (6.7)	1421 (4.9)	
Infectious Disease	248 (3.9)	1419 (4.9)	
Anesthesiology	269 (4.2)	903 (3.1)	
Emergency Medicine	268 (4.2)	794 (2.7)	
Gastroenterology	164 (2.6)	646 (2.2)	
Obstetrics and Gynecology	42 (0.7)	271 (0.9)	
Non-shock Specialty	178 (2.8)	871 (3.0)	
Other	232 (3.6)	1197 (4.1)	
Unknown	239 (3.7)	1309 (4.5)	

*PA* physician assistant, *NP* nurse practitioner

**Fig. 1:**
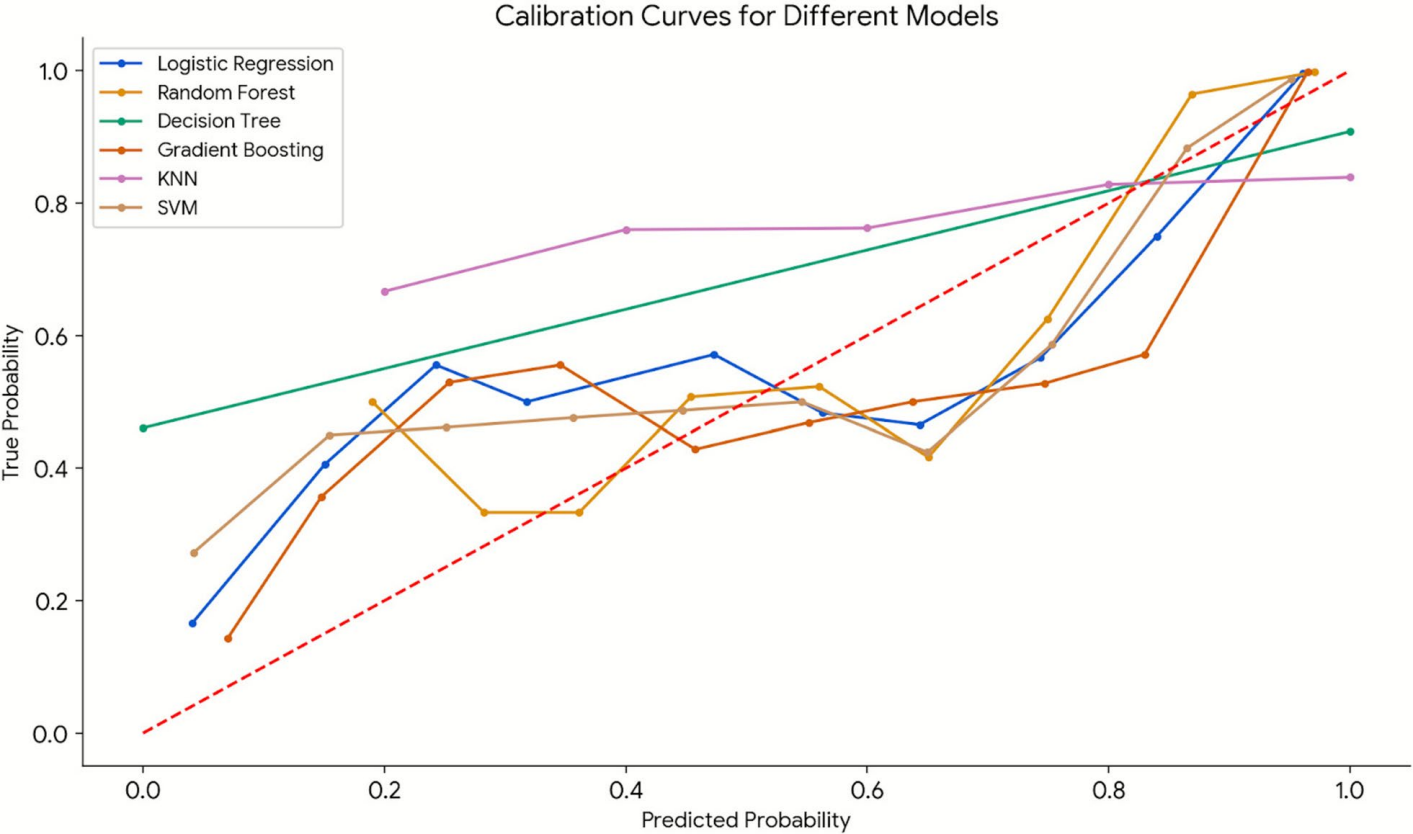
Calibration plots of machine learning model predicting never having complete inter-clinician diagnostic agreement of shock etiology within 9 acute care hospitals from 2018 to 2023

Six models were constructed to predict which patients never had complete inter-clinician diagnostic agreement of shock etiology and all were highly predictive (eTable 2). Logistic regression (Accuracy: 0.8597, F1 Score: 0.9117, AUC-ROC: 0.9257), Random Forest (Accuracy: 0.8658, F1 Score: 0.9201, AUC-ROC: 0.9255), and gradient boosting (Accuracy: 0.8515, F1 Score: 0.9138, AUC-ROC: 0.9227) were the three highest performing models. Model calibration curves for logistic regression, gradient boosting, and SVM were closest to the diagonal line suggesting that these models provide more reliable probability estimates (Fig. 1). The KNN model showed poor calibration, with its curve deviating significantly from the diagonal, indicating the model’s predicted probabilities were misaligned with the observed probabilities. The most important features in the Random Forest model predicting patients lacking inter-clinician diagnostic agreement on shock etiology were variations in shock etiology diagnoses (eFig. 2).

More lenient similarity cosign similarity thresholds (e.g., ≥ 0.95, ≥ 0.90) were tested to reflect real-world diagnostic convergence. A cosign similarity threshold of 0.95 demonstrated 16.3% of patients never had inter-clinician diagnostic agreement (eTables 3–6). A cosign similarity threshold of 0.90 demonstrated 16.1% of patients never had inter-clinician diagnosis agreement (eTables 7–10). These relaxed threshold sensitivity analyses demonstrated association stability and comparative model performance with a stringent threshold.

## Discussion

This study sought to characterize inter-clinician diagnostic agreement of shock etiology. Using a novel application of cosine similarity score we quantified 18.2% of shock patients never had complete inter-clinician diagnostic agreement. Furthermore, logistic regression and gradient boosting best predicted which patients would never have complete inter-clinician diagnostic agreement of shock etiology and were well calibrated. The findings of the current study fill a knowledge gap in quantifying rate of inter-clinician diagnostic agreement of shock etiology in actual clinical care never previously investigated.

This study builds upon previous literature which demonstrates that diagnostic agreement between different clinicians is poor [9, 22]. One study demonstrated that different specialty clinicians reached different conclusions, even when presented with the same clinical information, as a result of their discipline-specific frame of reference [23]. Furthermore, that patients without inter-clinician diagnostic agreement were more often Black was concerning. Racial difference in agreement was based on unadjusted comparisons and should be interpreted alongside social or contextual variables. Differences in diagnostic agreement may reflect a combination of illness severity, comorbidity burden, neighborhood environment and social determinants of health, as well as clinician documentation or communication patterns, rather than race alone. Currently, there is no standardized way for clinicians to share real-time diagnostic assessments, develop a shared understanding of shock etiology, or reach consensus on treatment [23–27]. The lack of consensus may delay diagnosis, and treatment, or as we saw lead to aggressive possibly unnecessary treatment ultimately thus increasing mortality.

The implications of this study underscore the critical need to design a systematic process to share information regarding clinicians' specialty-specific assessments to build a shared understanding of the shock etiology among all clinicians who care for shock patients. The association between diagnostic disagreement, more aggressive treatments and adverse clinical outcomes (e.g., reduced improvement in SOFA scores, increased mortality) was a significant finding that underscores the clinical consequences of fragmented diagnostic processes. Potential mechanisms include clinician-patient discordance, documentation variability, differential access from neighborhood deprivation, and heuristic bias. Although causal inference is limited by the design, the consistent associations and sensitivity analyses highlight a target group of patients where timely, structured inter-professional communication for reconciliation of the diagnosis may offer benefit to offer more narrow, targeted earlier treatment. The National Academy of Science has recognized that inter-professional communication is an essential component to the diagnostic process for patients with complex and highly lethal conditions, such as shock [28]. Identifying the patients more likely to experience clinician diagnostic disagreement,such as with our machine learning model, could be used in clinical practice to prompt inter-clinician communication mechanisms, to improve diagnostic agreement , optimize timely treatment, and ultimately reduce mortality. Prospective evaluation of this clinician decision support system is our next step.

This study has several limitations that are largely mitigated by a thorough study design. First, sampling bias was present as the study population encompassed patients from nine hospitals in a single integrated health system. However, the study population included thousands of patient encounters from many different ICUs across nine very different  acute care hospitals in a geographically varied state. Thus, the results are likely generalizable to a wide range of ICUs and hospitals. Second, selection bias may have been introduced as the study excluded patients where only one clinician note mentioned a diagnosis of shock because inter-clinician diagnostic agreement could only be calculated when at least two different clinicians documented a shock diagnosis. Therefore, the patients included in this study may be biased towards the most complex patients requiring many clinicians with high illness severity. This limits generalizability of our findings to less complex shock patients. The broader goal of this ongoing project is to identify patients who benefit from a real-time inter-professional communication intervention to enhance shock etiology diagnosis. One of the major concerns in our follow-up qualitative analysis and user-centered design study, were clinician alert fatigue. Excluding more straightforward cases of shock where only one clinician was involved therefore is a relative strength . External validation in other health systems to demonstrate generalizability to predict patients without inter-clinician diagnostic agreement is needed. Third, this study quantitatively analyzed inter-clinician diagnostic agreement among shock etiology but did not capture qualitative data regarding inter-clinician diagnostic agreement. A qualitative study investigating the barriers and facilitators of inter-professional communication in shock diagnosis is beyond the scope of this study and is forthcoming. Fourth, our approach has limitations inherent to regular expressions applied on clinical notes. Terminology varies by author, and documentation may lag behind clinical judgment. We mitigated this by: (1) using a strict lexicon with explicit string matches for each shock etiology and excluding negated statements; (2) requiring ≥ 2 independent clinician notes per patient-day to compute agreement; and (3) analyzing agreement over the first five hospital days rather than at a single time point. For septic shock, we used exact terms (“sepsis,” “septic,” “septic shock”) without negation and manually reviewed all occurrences to ensure context-sensitive language did not drive misclassification. A separate expert-consensus adjudication would be ideal, but our primary endpoint was agreement between clinicians as documented, not the correctness of any single diagnosis. Because the our pipeline captured the explicit labels clinicians recorded, consistent extraction across notes allowed valid estimation of relative agreement even if some absolute labels were imperfect. Construct validity supports this: patients without complete agreement had higher severity (SOFA > 15), more aggressive treatment (e.g., central venous access, Ccontinuous renal replacement, anticoagulation, bumetanide administration), slower antibiotic initiation, and higher ICU and in-hospital mortality—patterns expected when diagnostic consensus is difficult. These convergent findings suggest the regular expression labels reflected clinically meaningful discordance. Future work will include external validation in other health systems, expanded lexicons and abbreviation handling, and a sampled chart-review adjudication to quantify precision/recall across etiologies. Nonetheless, given our focus on measured agreement, system-wide scale (35,411 notes across nine hospitals), and the targeted manual review for septic shock, we believe the present findings are robust and informative.

Lastly, the study period overlapped with the COVID-19 pandemic which had a significant impact on outcomes for critically ill patients. However, the COVID-19 pandemic period was distributed evenly between patients with and without complete inter-clinician diagnostic agreement.

## Conclusion

This study is one of the first to quantify inter-clinician diagnostic agreement of shock etiology in clinical care. Patients without complete inter-clinician diagnostic agreement of shock etiology can be successfully predicted. These results highlight an opportunity to identify and improve the diagnostic process for shock patients. We believe this can be achieved through optimizing the inter-professional communication in the diagnostic process to improve the inter-clinician diagnostic agreement, treatment, and mortality of shock patients.

## Supplementary Information

Below is the link to the electronic supplementary material.

Supplementary file1 (DOCX 37 KB)

## References

[1] Holler JG, Henriksen DP, Mikkelsen S, et al. Shock in the emergency department; a 12 year population based cohort study. Scand J Trauma Resusc Emerg Med. 2016;24:87. 27364493 10.1186/s13049-016-0280-x PMC4929750

[2] Gitz Holler J, Jensen HK, Henriksen DP, et al. Etiology of shock in the emergency department: a 12-year population-based cohort study. Shock. 2019;51(1):60–7. 27984523 10.1097/SHK.0000000000000816 PMC6282680

[3] Jones AE, Aborn LS, Kline JA. Severity of emergency department hypotension predicts adverse hospital outcome. Shock. 2004;22(5):410–4. 15489632 10.1097/01.shk.0000142186.95718.82

[4] Jones AE, Tayal VS, Sullivan DM, Kline JA. Randomized, controlled trial of immediate versus delayed goal-directed ultrasound to identify the cause of nontraumatic hypotension in emergency department patients. Crit Care Med. 2004;32(8):1703–8. 15286547 10.1097/01.ccm.0000133017.34137.82

[5] Pruinelli L, Westra BL, Yadav P, et al. Delay within the 3-hour surviving sepsis campaign guideline on mortality for patients with severe sepsis and septic shock. Crit Care Med. 2018;46(4):500–5. 29298189 10.1097/CCM.0000000000002949 PMC5851815

[6] Davenport DL, Henderson WG, Mosca CL, et al. Risk-adjusted morbidity in teaching hospitals correlates with reported levels of communication and collaboration on surgical teams but not with scale measures of teamwork climate, safety climate, or working conditions. J Am Coll Surg. 2007;205(6):778–84. 18035261 10.1016/j.jamcollsurg.2007.07.039

[7] Brun-Buisson C, Doyon F, Carlet J, et al. Incidence, risk factors, and outcome of severe sepsis and septic shock in adults: a multicenter prospective study in intensive care units—French ICU Group for Severe Sepsis. JAMA. 1995;274(12):968–74. 7674528

[8] Paoli CJ, Reynolds MA, Sinha M, et al. Epidemiology and costs of sepsis in the United States-an analysis based on timing of diagnosis and severity level. Crit Care Med. 2018;46(12):1889–97. 30048332 10.1097/CCM.0000000000003342 PMC6250243

[9] Mehta S, Granton J, Lapinsky SE, et al. Agreement in electrocardiogram interpretation in patients with septic shock. Crit Care Med. 2011;39(9):2080–6. 21849822 10.1097/CCM.0b013e318222720e

[10] Danna G, Garg R, Buchheit J, et al. Prediction of intra-abdominal injury using natural language processing of electronic medical record data. Surgery. 2024. 10.1016/j.surg.2024.05.042. 10.1016/j.surg.2024.05.042 PMC11330356 38972771

[11] Gupta T, Puskarich MA, DeVos E, et al. Sequential organ failure assessment component score prediction of in-hospital mortality from sepsis. J Intensive Care Med. 2020;35(8):810–7. 30165769 10.1177/0885066618795400 PMC6669100

[12] Jones AE, Trzeciak S, Kline JA. The sequential organ failure assessment score for predicting outcome in patients with severe sepsis and evidence of hypoperfusion at the time of emergency department presentation. Crit Care Med. 2009;37(5):1649–54. 19325482 10.1097/CCM.0b013e31819def97 PMC2703722

[13] O’Leary KJ, Devisetty VK, Patel AR, et al. Comparison of traditional trigger tool to data warehouse based screening for identifying hospital adverse events. BMJ Qual Saf. 2013;22(2):130–8. 10.1136/bmjqs-2012-001102 23038408

[14] Hinchcliff M, Just E, Podlusky S, et al. Text data extraction for a prospective, research-focused data mart: implementation and validation. BMC Med Inform Decis Mak. 2012;12:106. 22970696 10.1186/1472-6947-12-106 PMC3537747

[15] Richie R, Bhatia S. Similarity judgment within and across categories: a comprehensive model comparison. Cogn Sci. 2021;45(8):e13030. 34379325 10.1111/cogs.13030

[16] Kind AJH, Buckingham WR. Making neighborhood-disadvantage metrics accessible - the Neighborhood Atlas. N Engl J Med. 2018;378(26):2456–8. 29949490 10.1056/NEJMp1802313 PMC6051533

[17] Vincent JL, de Mendonca A, Cantraine F, et al. Use of the SOFA score to assess the incidence of organ dysfunction/failure in intensive care units: results of a multicenter, prospective study: Working group on “sepsis-related problems” of the European Society of Intensive Care Medicine. Crit Care Med. 1998;26(11):1793–800. 9824069 10.1097/00003246-199811000-00016

[18] Singer M, Deutschman CS, Seymour CW, et al. The third international consensus definitions for sepsis and septic shock (Sepsis-3). JAMA. 2016;315(8):801–10. 26903338 10.1001/jama.2016.0287 PMC4968574

[19] Berger T, Green J, Horeczko T, et al. Shock index and early recognition of sepsis in the emergency department: pilot study. West J Emerg Med. 2013;14(2):168–74. 23599863 10.5811/westjem.2012.8.11546 PMC3628475

[20] Park SY, Park JE, Kim H, Park SH. Review of statistical methods for evaluating the performance of survival or other time-to-event prediction models (from conventional to deep learning approaches). Korean J Radiol. 2021;22(10):1697–707. 34269532 10.3348/kjr.2021.0223 PMC8484151

[21] Dietrich S, Floegel A, Troll M, et al. Random survival forest in practice: a method for modelling complex metabolomics data in time to event analysis. Int J Epidemiol. 2016;45(5):1406–20. 27591264 10.1093/ije/dyw145

[22] Beckmann U, Baldwin I, Hart GK, Runciman WB. The Australian incident monitoring study in intensive care: AIMS-ICU. An analysis of the first year of reporting. Anaesth Intensive Care. 1996;24(3):320–9. 8805886 10.1177/0310057X9602400304

[23] Liu P, Lyndon A, Holl JL, et al. Barriers and facilitators to interdisciplinary communication during consultations: a qualitative study. BMJ Open. 2021;11(9):e046111. 10.1136/bmjopen-2020-046111 PMC8413943 34475150

[24] Birkmeyer NJ, Finks JF, Greenberg CK, et al. Safety culture and complications after bariatric surgery. Ann Surg. 2013;257(2):260–5. 23047607 10.1097/SLA.0b013e31826c0085

[25] Alvarez G, Coiera E. Interdisciplinary communication: an uncharted source of medical error? J Crit Care. 2006;21(3):236–42. 16990088 10.1016/j.jcrc.2006.02.004

[26] Lingard L, Espin S, Whyte S, et al. Communication failures in the operating room: an observational classification of recurrent types and effects. Qual Saf Health Care. 2004;13(5):330–4. 15465935 10.1136/qshc.2003.008425 PMC1743897

[27] Janczewski LM, Chandrasekaran A, Abahuje E, et al. Barriers and facilitators to end-of-life care delivery in ICUs: a qualitative study. Crit Care Med. 2024;52(6):e289–98. 38372629 10.1097/CCM.0000000000006235 PMC11218910

[28] Ball JR, Balogh E. Improving diagnosis in health care: highlights of a report from the National Academies of Sciences, Engineering, and Medicine. Ann Intern Med. 2016;164(1):59. 26414299 10.7326/M15-2256

