# Supplementary material for: Inter-clinician diagnostic agreement of shock etiology: a multicenter observational study
Source: Health Inf Sci Syst. 2026 Jan 12;14(1):27. doi: 10.1007/s13755-025-00423-w (PMC12796023; doi:10.1007/s13755-025-00423-w)
Supplement: Supplementary file 1 — Supplementary file1 (DOCX 37 KB) [file 13755_2025_423_MOESM1_ESM.docx]

**Supplemental Digital Content**

**eFigure 1.** **Consort Diagram of** **Shock Patients with at Least Two Clinician Diagnosing Shock Etiology Captured in Electronic Health Record Clinician Notes in 9 Acute Care Hospitals from 2018-2023.**

**eTable 1. List of Antibiotics Administered.**

**eFigure 2.** **Feature Importance in Prediction of Never Having Complete Inter-clinician Diagnostic Agreement of Shock Etiology within 9 Acute Care Hospitals from 2018-2023**.

**eFigure 1.** **Consort Diagram of** **Shock Patients with at Least Two Clinician Diagnosing Shock Etiology Captured in Electronic Health Record Clinician Notes in 9 Acute Care Hospitals from 2018-2023.**

830 patient encounters excluded due to multiple patient encounters for the same patient (only the last encounter was included)

45,740 patient encounters excluded for having only 1 clinician mention shock in a note from day 0 to day 4 of the ICU hospital stay

Inclusion criteria:

- Years 2018-2023
- Diagnosed with shock (i.e. systolic BP <90 mmHg)
- At least 18 years old
- Evidence of at least 1 new organ failure by SOFA (Sequential Organ Failure Assessment)

Patient encounters assessed for eligibility among 9 acute care hospitals in a single health system

8,132 patient encounters included

53,872 patient encounters identified

7,302 unique patient encounters included

**eTable 1. List of Antibiotics Administered.**

| *Name of Antibiotic* |
| --- |
| Vancomycin |
| Ceftriaxone |
| Piperacillin-Tazobactam |
| Ertapenem |
| Amoxicillin-Clavulanate |
| Linezolid |
| Meropenem |
| Cefepime |
| Metronidazole |
| Amikacin |
| Colistin |

**eFigure 2.** **Feature Importance in Prediction of Never Having Complete Inter-clinician Diagnostic Agreement of Shock Etiology within 9 Acute Care Hospitals from 2018-2023**.
